# Community-based e-health program on self-care and cognition of older adults: multi-site cluster-controlled trial

**DOI:** 10.1093/geroni/igaf122.2288

**Published:** 2025-12-31

**Authors:** Vivien Xi Wu, Xin Yi Yap, Poh Choo Tan, Wai San, Wilson Tam

**Affiliations:** National University of Singapore, Singapore, Singapore; National University of Singapore, Singapore, Singapore; Changi General Hospital, Singapore, Singapore; National University of Singapore, Singapore, Singapore

## Abstract

**Objective:**

The growing prevalence of chronic diseases among the older population has become a global public health concern. This study aims to examine the effects of a 12-week Community-based eHealth Program (CeHP) in enhancing the self-care, cognition and health outcomes for community-dwelling older adults with chronic diseases. Design, setting and participants: A non-randomized cluster-controlled trial was conducted. Participants were recruited from 11 Active Ageing Centers (AACs) affiliated with Community Health Posts located in the Eastern region of Singapore. Six AACs served as the intervention group where five AACs served as control. The CeHP consisted of seven in-person sessions at the AACs and five virtual sessions through Care4Senior App. The outcome measures included self-care abilities of chronic illness, cognition, health literacy, healthy ageing practice, empowerment, social support and physical function at baseline, immediate post-intervention and 6-month follow-up. Repeated measure analysis was conducted using linear mixed effect models.

**Results:**

A total of 108 participants (n = 61 in intervention; n = 47 in control) completed the study. The CeHP intervention resulted in significant effect on the cognition among community-dwelling older adults with chronic diseases. However, no significant difference was observed on other health outcomes.

**Conclusion/Implications:**

eHealth solutions are gaining prominence in improving quality of care and changing how individuals manage their health. However, disparities in digital access, technological proficiency and eHealth literacy continue to impact on the vulnerable older population. Further research could continue to explore eHealth interventions and support system to minimize the barriers to eHealth uptake among the older adults.

